# Analysis of plant-derived miRNAs in animal small RNA datasets

**DOI:** 10.1186/1471-2164-13-381

**Published:** 2012-08-08

**Authors:** Yuanji Zhang, B Elizabeth Wiggins, Christina Lawrence, Jay Petrick, Sergey Ivashuta, Greg Heck

**Affiliations:** 1 Chesterfield Village Research Center, Monsanto Company, 700 Chesterfield Parkway, North Chesterfield, MO, 63017, USA; 2 St. Louis – World Headquarters, Monsanto Company, 800 North Lindbergh Blvd, St. Louis, MO, 63167, USA

**Keywords:** Plant miRNA, Animal small RNA datasets, RNAi, miR168, Aphid, Corn ear worm, Corn rootworm, Fall army worm, Silkworm

## Abstract

**Background:**

Plants contain significant quantities of small RNAs (sRNAs) derived from various sRNA biogenesis pathways. Many of these sRNAs play regulatory roles in plants. Previous analysis revealed that numerous sRNAs in corn, rice and soybean seeds have high sequence similarity to animal genes. However, exogenous RNA is considered to be unstable within the gastrointestinal tract of many animals, thus limiting potential for any adverse effects from consumption of dietary RNA. A recent paper reported that putative plant miRNAs were detected in animal plasma and serum, presumably acquired through ingestion, and may have a functional impact in the consuming organisms.

**Results:**

To address the question of how common this phenomenon could be, we searched for plant miRNAs sequences in public sRNA datasets from various tissues of mammals, chicken and insects. Our analyses revealed that plant miRNAs were present in the animal sRNA datasets, and significantly miR168 was extremely over-represented. Furthermore, all or nearly all (>96%) miR168 sequences were monocot derived for most datasets, including datasets for two insects reared on dicot plants in their respective experiments. To investigate if plant-derived miRNAs, including miR168, could accumulate and move systemically in insects, we conducted insect feeding studies for three insects including corn rootworm, which has been shown to be responsive to plant-produced long double-stranded RNAs.

**Conclusions:**

Our analyses suggest that the observed plant miRNAs in animal sRNA datasets can originate in the process of sequencing, and that accumulation of plant miRNAs via dietary exposure is not universal in animals.

## Background

Small RNAs (sRNAs) are a key component of RNA-based regulatory system with basic regulatory mechanisms being conserved in eukaryotes. Plant tissues contain significant quantities of sRNAs [1], which are usually processed from long double-stranded RNA (dsRNA) precursors by RNase III enzymes. These sRNAs can be divided into two major categories: small interfering RNAs (siRNAs) and microRNAs (miRNAs). Although a smaller proportion of the total sRNA population, miRNAs are less diverse and particular miRNAs can dominate the stoichiometry amongst individual sRNA species [2,3].

By ingesting plant material, animals are exposed to considerable amount of RNA including sRNAs. Given the diverse number of sequences present in sRNA populations, complementary matches to transcripts in animal consumers are readily identifiable [1]. Such complementary sRNAs are unlikely to be involved in heterologous regulation of gene expression in animals. In order to achieve any impact on gene expression in a consuming organism, sRNAs would need to be absorbed and distributed in biologically relevant quantities within the cells of animal tissues and organs. There are a number of key biological barriers to oral activity of ingested nucleic acids, including the harsh pH environment of the stomach and RNA-destructive condition of the gastrointestinal (GI) tract where nucleases and associated microbiota are present [4]. Any nucleic acids that are absorbed must also escape nucleases in cellular compartments and in the bloodstream thus limiting any potential activity of exogenous RNA molecules [5]. Furthermore, nucleic acid therapeutics usually lack systemic activity following intravenous injection due to their rapid filtration by the kidney and renal elimination [6,7]. For these reasons, delivery of oligonucleotide therapeutics does not occur orally but is administered locally or systemically with the use of specialized lipophilic delivery vehicles and synthetic modifications to native RNA structure.

A recent publication [8] suggests that some plant miRNAs can pass through the animal GI track and enter the circulatory system and various organs presumably protected by association with microvesicles. Interestingly, mature miR168 was one of the plant miRNAs detected at the highest level in mice fed with raw rice. To address the question how widespread this phenomenon is in the animal kingdom, we conducted an analysis of public sRNA datasets from various vertebrate and invertebrate animals for presence of plant miRNA sequences. Surprisingly, the miR168 sequence was detected as the predominant or sole plant miRNA in animal datasets, including insect examples from different phylogenetic lineages, representing diverse digestive anatomy and physiology. Publically available insect sRNA datasets were limited, however, so we initiated controlled feeding experiments in readily accessible, lab-reared lepidopteran and coleopteran insect representatives to examine if miRNA uptake is a general faculty of insects. *Diabrotica virgifera vergifera* LeConte (western corn rootworm, WCR) was included in the analysis since its responsiveness to ingested plant-produced dsRNA was previously established [9]. In addition to the public data analysis results, here we also describe the observations of miRNA uptake as a result of plant feeding in selected insects.

## Results

### Computational analysis of animal public sRNA datasets identified plant derived miRNAs

**Figure 1:**
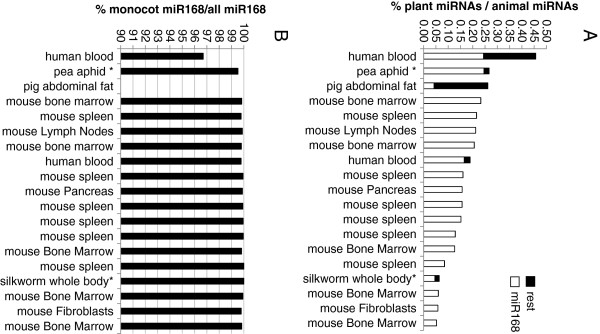
**Monocot miR168 is over-represented in detected plant miRNAs in 19 animal sRNA datasets.A**. Relative proportion of miR168 vs. other plant miRNA families observed in sRNA datasets. **B**. Relative abundance of monocot miR168 sequence observed. Asterisk indicates insect samples.

We examined the prevalence of identifiable plant miRNAs in sRNA datasets derived from various animal sources with different sampling techniques and experimental and analytical methodologies. Of 83 animal sRNA public datasets used for analysis, 63 (including 5 datasets from human and mouse cultured cell lines) had at least one sequence that had perfect identity to a known plant miRNA (Additional file 1 and Additional file 2). In 19 datasets, plant miRNA reads were at least 0.050% of the total animal miRNA reads (Table 1) for samples from human (2 datasets), mouse (14), pig (1), pea aphid (1), and silkworm (1) (Figure 1). The most abundant plant miRNA sequence observed in any instance is numerically not within the top 10 most abundant endogenous animal miRNA. Significant variation exists in the number of observed plant miRNAs even in datasets from the same tissue or experimental repetition. For example, 2016 out of 3,989,601 raw reads from SRR042446 (sample GSM539838, mouse mature B cells, spleen replicate 1) match to plant miRNAs, while none were observed in 9,669,987 reads from SRR042447 (sample GSM539839, mouse mature B cells, spleen replicate 2). The highest observed ratio of plant miRNAs/animal miRNAs is 0.456%, which is 10 times lower than a figure of ~5% reported by Zhang et al. [8].

**Table 1:** Animal small RNA datasets where significant amount of plant miRNAs were detected

**SRA Run ID**	**Organism**	Source*	**miRNAs (animal + plant)**	**Plant miRNAs**		**Most abundant plant miRNA family**				
				**Reads**	**% of animal miRNAs**	**Family**	**Reads**	**% of plant miRNAs**	**% of animal miRNAs**	**Rank in animal miRNA families**
SRR039190	human	blood	1175650	5342	0.456	miR168	2856	53.5	0.244	42
SRR036085	pea aphid	whole insect	692109	1841	0.267	miR168	1695	92.1	0.246	18
SRR080701	pig	abdominal fat	2572468	6709	0.261	miR535	2835	42.3	0.110	36
SRR042444	mouse	bone marrow	3620895	8412	0.233	miR168	8411	100.0	0.233	15
SRR042463	mouse	spleen	2092533	4500	0.216	miR168	4499	100.0	0.215	24
SRR042454	mouse	lymph nodes	2019824	4274	0.212	miR168	4274	100.0	0.212	14
SRR042443	mouse	bone marrow	3759698	7738	0.206	miR168	7732	99.9	0.206	18
SRR039191	human	blood	2195526	4153	0.190	miR168	3627	87.3	0.166	28
SRR042448	mouse	spleen	2845389	4549	0.160	miR168	4548	100.0	0.160	16
SRR042481	mouse	pancreas	2358808	3693	0.157	miR168	3693	100.0	0.157	23
SRR042445	mouse	spleen	3165427	4952	0.157	miR168	4951	100.0	0.157	15
SRR042451	mouse	spleen	2238211	3397	0.152	miR168	3397	100.0	0.152	22
SRR042467	mouse	spleen	3308658	4264	0.129	miR168	4262	100.0	0.129	23
SRR042456	mouse	bone marrow	2645549	3344	0.127	miR168	3344	100.0	0.127	32
SRR042446	mouse	spleen	2367601	2016	0.085	miR168	2015	100.0	0.085	24
SRR035544	silkworm	whole body	1111992	705	0.063	miR168	508	72.1	0.046	28
SRR042462	mouse	bone marrow	2133939	1279	0.060	miR168	1276	99.8	0.060	37
SRR042475	mouse	embryonic fibroblasts	2438884	1420	0.058	miR168	1420	100.0	0.058	51
SRR042457	mouse	bone marrow	3370053	1777	0.053	miR168	1777	100.0	0.053	31

*Please visit http://www.ncbi.nlm.nih.gov/sra/ for detailed descriptions of source tissues and cell types.

For all the datasets analyzed, reads mapping to plant miRNAs were mostly or exclusively miR168, except for the pig abdominal fat dataset (SRR080701) where the most abundant plant miRNA family is miR535, which accounts for 42.3% of total plant miRNAs within the plant-specific miRNAs observed in that sample (Table 1, Figure 1A). The second and third most abundant miRNAs in the pig abdominal fat dataset are miR156 and miR168, with 1584 and 1085 reads, respectively (Additional file 2). Interestingly, the miR168 sequence in pig is predominately UCGCUUGGUGCAGGUCGGGAA, which is found in dicots such as *Arabidopsis* (ath-miR168a and b), soybean (gma-miR168), and *Brassica napus* (bna-miR168). In contrast, predominate miR168 sequence in other datasets is UCGCUUGGUGCAGAUCGGGAC, which is only found in monocots such as rice (osa-miR168a), corn (zma-miR168a & b), and *Sorghum bicolor* (sbi-miR168) (Figure 1B).

**Figure 2:**
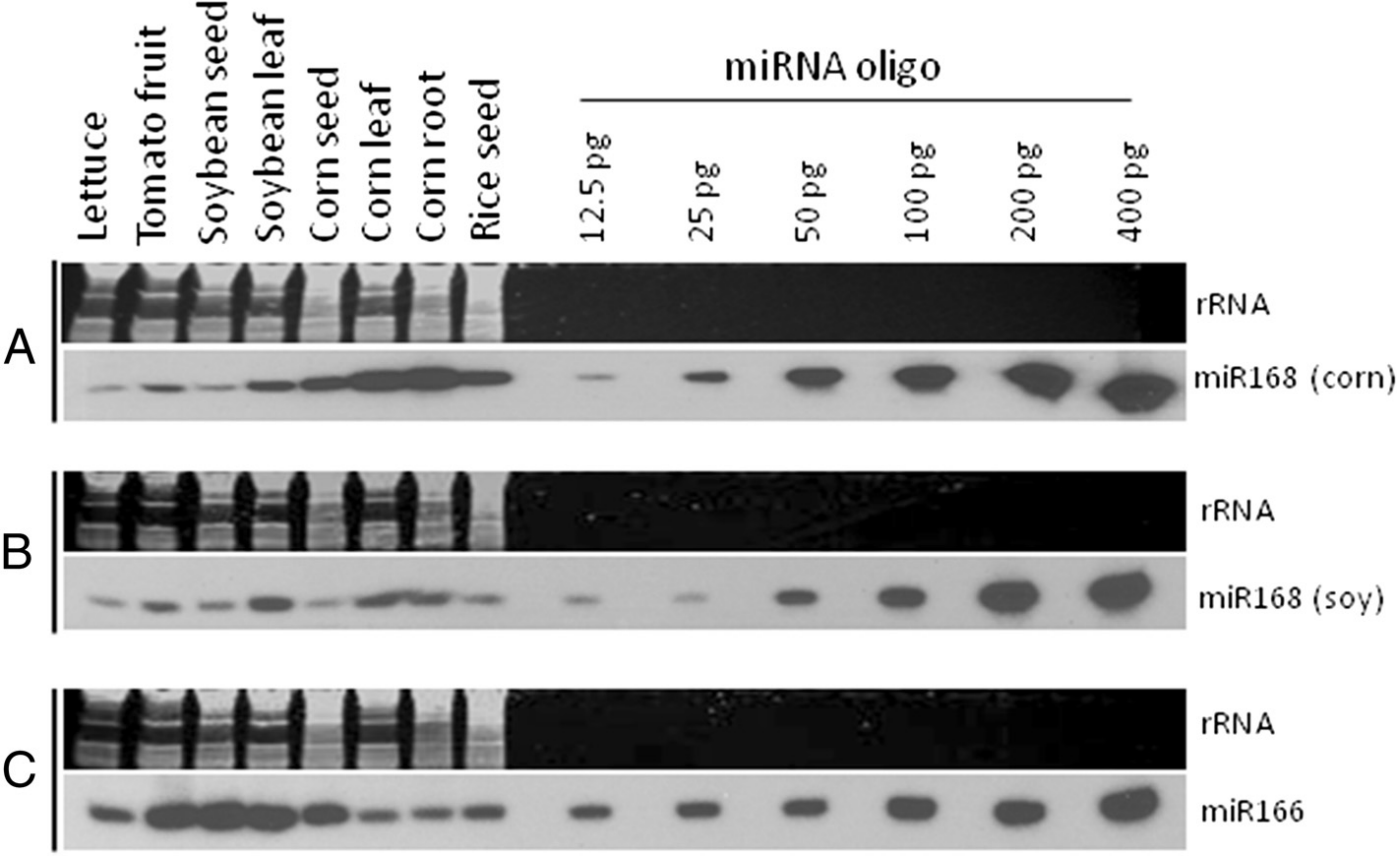
**Northern blot analysis of accumulation of sRNAs in plant organs.** RNA from tissues of fresh lettuce leaf, green tomato fruit, mature soybean seed, soybean leaf, mature corn seed, corn leaf, corn root, dehulled rice grain (*Oryza sativa* spp. *japonica* cv. Nipponbare) was probed for corn miR168 sequence (Panel **A**); soybean miR168 sequence (Panel **B**), or miR166 (Panel **C**). Twenty-one base synthetic RNA oligonucleotides were included on the gel at various concentrations (12.5-400 pg) for semi-quantitative comparison.

Grain is likely the route of exposure to plant miRNAs for many domesticated animals, so we evaluated the miRNA abundance from the seed of rice, corn and soybean, using our sRNA sequence data. miR168 is highly expressed in corn kernel and rice grain, but is not the most abundant miRNA in these plants. In soybean seed, miR168 ranks below 20^th^ within the steady-state abundance of miRNAs (Additional file 3). This observation suggests that the presence of miR168 as a dominant plant miRNA in animal sRNA datasets from animal tissues cannot be explained simply as a reflection of its relative miRNA abundance in plants. sRNA northern blot analysis of select miRNAs from different plant sources that are variously parts of animal diets corroborates our sequencing result that miR168 is equal to or less abundant than other plant miRNAs in soybean, rice and corn seed (Figure 2).

To investigate whether the observed plant miRNAs are potentially encoded by animal genomes, we compared some of the most abundant plant miRNAs in public animal sRNA datasets against the National Center for Biotechnology Information (NCBI) nucleotide sequence database (NT), but excluded plant sequences. The plant miRNAs used in the search include miR156, miR166, miR167, miR168, miR535 and miR3522. We did not observe any perfect matches between plant miRNAs and non-plant sequences from NT dataset, which includes sequences from animals, fungi, bacteria, and viruses. Thus, any plant miRNAs detected in animal datasets are likely not to be derived from genomes of the host, pathogens, or microbiota associated with the animals.

### Analysis of plant miRNA in insects

**Figure 3:**
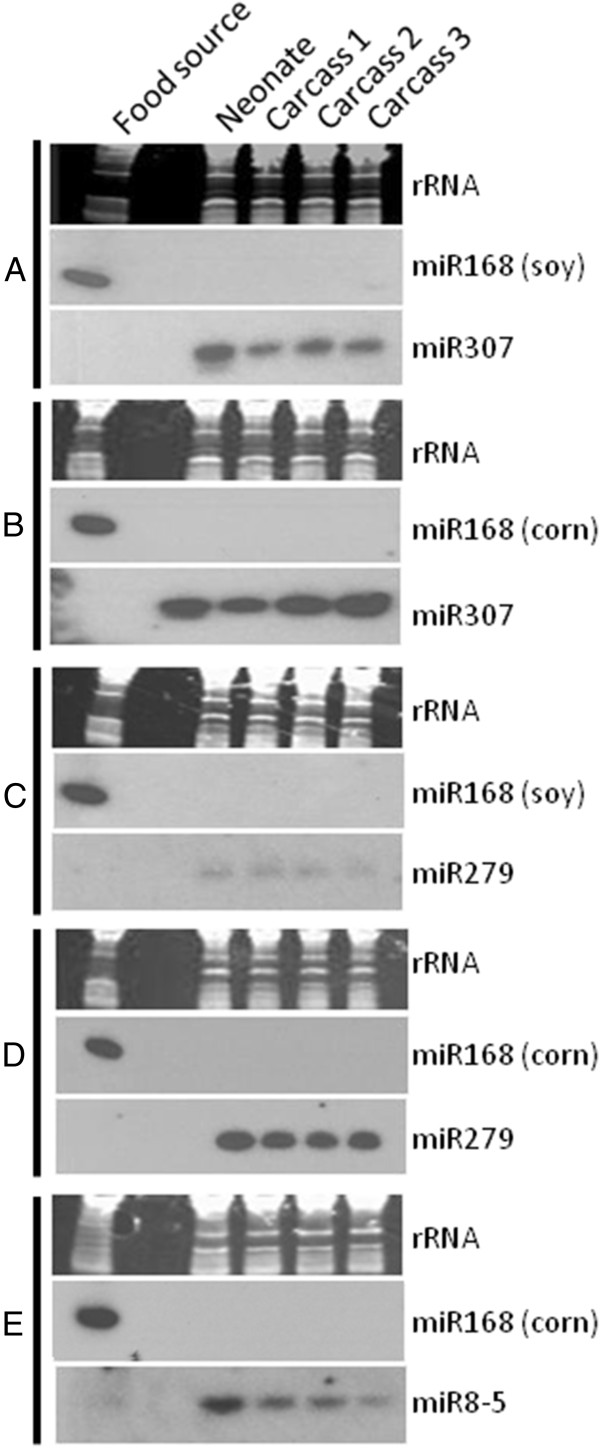
**Northern blot analysis of sRNAs in insect samples.** FAW and CEW neonates were fed on soybean leaf (Panels **A** and **C**) or corn leaf (Panels **B** and **D**) until the fifth instar. WCR neonates were fed on corn roots (Panel **E**) until the third instar. Three replicates of insect carcass RNA was probed for uptake of miR168 or endogenous insect control miRNAs (miR-307, miR-279, and miR-8-5p). Ribosomal RNA and insect-specific miRNAs indicate comparable loading of RNA among replicates.

In order to validate the direct impact of a food source upon the selective accumulation of miR168 in insects, we conducted controlled feeding experiments and sRNA northern blot analysis to detect miR168 and other sRNAs in *Helicoverpa zea* Boddie (corn earworm, CEW), *Spodoptera frugiperda* J.E.Smith (fall armyworm, FAW) and WCR feeding on natural plant tissues. For the lepidopteran larvae, CEW and FAW neonates were each split into two groups and fed on one of two plant food sources that have known miR168 nucleotide sequences that are distinguished by two nucleotide differences (soybean: UCGCUUGGUGCAGGUCGGGAA; corn: UCGCUUGGUGCAGAUCGGGAC). Larvae were grown to the third or fifth instar on the selected food source before sampling for analysis. Although miR168 was easily observed in the plant samples used for feeding (Figure 2), northern blot analysis did not reveal detectable miR168 signal in tested insects (Figure 3). Blots were stripped and re-probed with insect-specific miRNAs (miR-307, miR-279, and miR-8-5p), indicating the consistent quality of sRNA prepared from insects. To eliminate the possibility of non-detection due to sensitivity limits of northern blot analysis, we performed deep sequencing on the samples to evaluate the possibility of low level presence of plant-derived miRNAs. Three non-feeding neonate plus 15 corn- or soybean-fed instar insect libraries were sequenced in one multiplexed run (Run 1) with 13 plant libraries. Plant miRNAs were detected in all insect libraries including neonate libraries (Table 2). Unlike publically available datasets, the predominant detected plant miRNA for most insect libraries sequenced, including corn-fed insect libraries, is miR1507, which is not found in monocots such as corn. miR168 is the dominant miRNA in only one insect library (Feeding 6, CEW fed on corn leaf, replicate 3 in Table 2). Even in this instance, the number of reads mapped to miR168 is moderate. For all other insect libraries, miR168 represents no more than the seventh most abundant number of reads that map to plant miRNAs (Table 2). Presence of miR168 comparable to that observed in pea aphid and silkworm from NCBI was not observed in CEW, FAW, and WCR.

**Table 2:** Sequencing result and reads mapped to plant miRNAs from insect and plant sRNA libraries in Run 1

**Sample ID**	**Organism**	**Description**	**Raw reads**	**plant miRNA**	%plant/all**	**top plant miRNA**	**reads**	**miR168 reads (rank)**
lettuce 1 ~ 10*	Lettuce	10 individual lettuce samples	151549912	975866	97.558	396	589392	70430 (2 ~ 4)
diet 1	Corn	leaf	15015721	368664	98.911	396	95687	8952 (9)
diet 2	Soybean	leaf	17723129	1775205	99.918	159	446258	13467 (15)
diet 3	Corn	root	9890019	71668	82.096	156	29703	4533 (4)
feeding 1	WCR	neonate	16544248	1045	0.099	1507	207	19 (10)
feeding 2	CEW	neonate	14016629	790	0.073	1507	205	41 (6)
feeding 3	FAW	neonate	15429925	859	0.061	1507	403	12 (7)
feeding 4	CEW	diet corn leaf, carcass 5^th^ instar rep1	13469808	165	0.012	1507	55	1 (14)
feeding 5	CEW	diet corn leaf, carcass 5^th^ instar rep2	19188383	872	0.418	1507	329	9 (10)
feeding 6	CEW	diet corn leaf, carcass 5^th^ instar rep3	13020144	210	0.019	168	43	43 (1)
feeding 7	FAW	diet corn leaf, carcass 5^th^ instar rep1	14496854	648	0.077	1507	162	32 (7)
feeding 8	FAW	diet corn leaf, carcass 5^th^ instar rep2	23688055	592	0.017	1507	182	13 (8)
feeding 9	FAW	diet corn leaf, carcass 5^th^ instar rep3	8346959	305	0.028	1507	143	2 (14)
feeding 10	FAW	diet soy leaf, carcass 5^th^ instar rep1	13583831	903	0.229	1507	263	27 (9)
feeding 11	FAW	diet soy leaf, carcass 5^th^ instar rep2	20007178	1065	0.310	1507	231	33 (8)
feeding 12	FAW	diet soy leaf, carcass 5^th^ instar rep3	16090620	2773	0.298	159	719	28 (12)
feeding 13	WCR	diet corn root, carcass 3^rd^ instar rep1	23799773	509	0.064	1507	177	10 (8)
feeding 14	WCR	diet corn root, carcass 3^rd^ instar rep2	20263174	400	0.051	1507	134	6 (10)
feeding 15	WCR	diet corn root, carcass 3^rd^ instar rep3	18023806	242	0.038	1507	70	7 (7)
feeding 16	CEW	diet soy leaf, carcass 5^th^ instar rep1	13292508	2947	0.361	3522	671	12 (14)
feeding 17	CEW	diet soy leaf, carcass 5^th^ instar rep2	17506112	1744	0.110	3522	574	13 (13)
feeding 18	CEW	diet soy leaf, carcass 5^th^ instar rep3	failed					

*Numbers in the row are the totals of 10 lettuce libraries.

**This is the ratio of plant miRNA reads over all miRNA reads. For plant datasets all miRNAs include contaminated animal miRNAs.

## Discussion

### Monocot miR168 observed is disproportionately abundant in the public animal datasets and may be adventitious in some circumstances

Monocot miR168 is the dominant or singular plant miRNA observed in most analyzed public sRNA animal datasets. This result is unlikely due to trivial contamination of animal sRNA libraries with plant material, because in any plant tissue, multiple plant miRNAs are expressed and miR168 in many cases is not the most abundant plant miRNA (e.g., Figure 2 and Additional file 3). We hypothesize that the plant miRNA abundance detected in animal tissue datasets should reflect the distribution of miRNAs in plant tissues unless miR168 undergoes preferential uptake or stabilization in animals, or that alternatively there is active discrimination against more abundant plant miRNAs. The public datasets where miR168 sequence is over-represented in detected plant miRNAs are from insects, chicken and mammals. These animals belong to distinct lineages with diverse digestive anatomy and physiology. This could implicate a special property of miR168 and select additional miRNAs to be preferentially stable and/or have particular associations with other factors that protect and shepherd them through the GI tract and into distal organs. However, the appearance of miR168 does not align with the experimental setup in all cases. For instance, over 99% of miR168 from pea aphid and 100% of miR168 from silkworm datasets are of monocot origin (Figure 1). These two insects populations were, however, reared on the dicot plants broad bean (*Vicia faba*) and mulberry (*Morus alba*), respectively [10,11]. While neither of these dicot food sources have publically accessible sRNA datasets to confirm fortuitous identity to the monocot version of miR168, datasets are available for soybean and *Medicago truncatula* (other species within the same sub-family, Faboideae, of the Fabaceae that includes broad bean as a member) support the likely conservation of dicot miR168 sequence in broad bean. Furthermore, our insect feeding experiments did not reveal any specific/preferential accumulation of miR168 in insects fed on a plant diet containing miR168. Combined, our observations suggest that the observed predominant monocot miR168 sequence is present as a result of contamination from a non-plant source.

### Possibilities of contamination from biological sources

For the 19 public animal sRNA datasets with numerous sequence reads that matched to plant miRNAs (Table 1, Figure 1), if we exclude the datasets where plant miRNAs are overwhelmingly monocot miR168, then only two (SRR039190 from human blood and SRR080701 from pig abdominal fat) have significant levels of plant miRNAs other than miR168. There are two possibilities for the presence of plant miRNAs in these two datasets: diet and from plant material contamination. If the former is true, then it is hard to explain why plant miRNAs are not detected at significant levels in most other datasets where animals are feeding on plant materials. In this manuscript we have analyzed six pig sRNA datasets (Additional file 2). Datasets SRR080702 (longissimus dorsi muscle) and SRR080700 (liver) are from the same pig individual as the plant miRNA-rich dataset SRR080701 [12], but have much fewer reads matched to plant miRNAs (the ratio of plant miRNAs/animal miRNAs are 0.001 and 0.023% for SRR080702 and SRR080700, respectively, comparing to 0.261% for SRR080701). The datasets SRR080698 and SRR080701 are from the same pig tissue, but from two individuals who form the full-sib F2 female pair in the experiment [12]. The ratio for SRR080698 is nearly 10 times lower (at 0.032%) than that for SRR080701 (Additional file 2).

In next-generation high-throughput sequencing technologies, multiplexing is used such that a number of libraries are sequenced together in one run. These libraries can be from different samples, experiments and/or organisms, and cross-contamination could arise in sequencing in such studies. Contamination may also occur in earlier steps before sequencing, such as library preparation. Next-generation high-throughput sequencing has been much more widely used in animal studies than in plant studies. According to the NCBI Sequence Read Archive (SRA) database, there are 61,809 archived Metazoa experiments versus 6,713 for Viridiplantae. As such, public animal sRNA datasets analyzed in this manuscript are more likely to be contaminated by other animal samples than by plant samples. To test this hypothesis, we searched public human sRNA datasets for non-human animal miRNAs to look for further evidence of adventitious sequences. A significant number of reads mapping to non-human animal miRNAs but not to known human miRNA or the human reference genome were detected in all human datasets, including datasets for cultured cell lines (Additional file 4). The most abundant non-human/mammalian animal-derived miRNAs detected in the human datasets were from fish, insects, chicken and frogs. Since by-products from these non-mammalian sources are typically not utilized in human cell culture media, the presence of miRNAs from these species in human cultured cell datasets is very likely from contamination versus dietary contribution. This result indicates that contamination should be considered during data interpretation when analyzing sRNA dataset from different sources.

We noted the occurrence of sequencing-derived presence of plant sRNAs within our own sequencing Run 1, where insect and lettuce libraries were multiplexed. One lettuce-specific sequence that produced numerous sRNAs in lettuce libraries also appeared in the insect data. A low number of raw reads from all insect libraries map to the lettuce specific sequence (Additional file 5). For the failed insect library which was successfully re-sequenced in Run 2, where no lettuce libraries were sequenced, none of its raw reads map to the lettuce-specific sequence. This indicates that cross contamination of the multiplexed libraries has occurred.

In addition, miR168 detected from corn and soybean-fed insect libraries in Run 1 has a mixture of dicot and monocot miRNA sequences, i.e., both dicot and monocot miR168 sequences are detected in corn-fed insects and in soybean-fed insects (data not shown). Since in the same run there are 10 lettuce leaf libraries, one soybean leaf library and two corn libraries, it is very likely that monocot miR168 in soybean-fed insects results from contamination from the corn library and the dicot miR168 in corn-fed insects is from soybean/lettuce libraries.

If contamination contributes to the observation of miR168 in our WCR, CEW, and FAW datasets, then other plant miRNAs should be similarly affected. Therefore, we also compared plant miRNA expression patterns between insect and source plant libraries, and between all insect and all plant libraries in Run 1. As shown in Additional file 6, plant miRNA abundance distribution for insects fed on corn is different from that of the source corn tissues, because there are several relatively highly abundant plant miRNAs in insect datasets but are very low or absent in the corn datasets. In contrast, plant miRNA expression patterns are similar between the group including plant miRNAs from all plant library reads and the group including plant miRNAs from all insect library reads in the run, strongly suggesting that all plant libraries within the multiplexed run contribute to the observed plant miRNAs in insect datasets. Since in the run most plant libraries are from dicot leaves (lettuce and soybean) and they share similar miRNA expressions, it is not surprising that the insects fed on soybean leaf has a similar plant miRNA expression pattern to that of its source tissue, i.e., soybean leaf (Additional file 6).

## Conclusions

In summary, our analysis suggests that plant miRNAs observed in some public animal sRNA datasets and our own insect feeding experiment sequence data may be artifactual due to sequencing methodology, and that accumulation of plant miRNAs via diet is not a common faculty among animals. Additional investigation is needed to address discrepancies observed in different studies, determine if adventitious presence accounts for all observations, ascertain the nature of enrichment where it appears to have occurred, and verify whether or not plant sRNA accumulation and circulation occurs at functional levels in some animal species.

## Methods

### Public sRNA datasets and analysis

A list of sRNA datasets was obtained by querying from NCBI SRA database (http://www.ncbi.nlm.nih.gov/sra). A total of 83 datasets derived from tissues of whole organisms were chosen from human, mouse, monkey, pig, chicken and four insects fed on plants. Datasets from cultured human and mouse cells were also selected as comparators since the datasets should not be directly affected by plant sRNAs resulting from dietary exposure. Datasets tissue source and raw number of sequence reads are summarized in Additional file 1 and Additional file 2.

The raw reads of the datasets were compared with all miRNA sequences from miRBase v17 at http://www.mirbase.org[13] using SHRiMP2 [14] as the mapping tool. A perfect match for the entire length of a given miRNA was required. Raw reads that mapped to plant miRNAs were also mapped to the sample source organism genome if available. Any reads with ≥ 20 nt perfect match to the genome was considered derived from the animal genome and were excluded from plant miRNA match count.

### Insect bioassays and northern blot analysis of sRNAs

FAW and CEW neonates <18 hours old were placed on fresh detached soybean (*Glycine max*) or corn (*Zea mays*) leaves and allowed to feed at 27°C, 60% relative humidity. Leaf tissue was replenished as needed. When the insects reached the fifth instar, carcass material (GI tract dissected and removed) from 3 insects was harvested and pooled for each replicate. WCR neonates were applied to corn roots and allowed to feed until the third instar, at which point carcass material was harvested from 10 larvae per replicate.

A plant organ panel for northern blot analysis included fresh lettuce leaf (*Lactuca sativa*), green tomato fruit (*Solanum lycopersicum*), mature soybean seed, soybean leaf, mature corn seed, the third vegetative (V3) corn leaf, corn root (hybrid), and dehulled rice grain (*Oryza sativa* spp. *japonica* cv. Nipponbare). Corn and soybean leaf samples were split and used for northern blot analysis, as well as sequencing and feeding studies.

Total RNA was extracted from plant and insect tissues using TRIzol reagent (Invitrogen). RNA oligos were obtained from Integrated DNA Technologies. Ten micrograms of total RNA and various amounts of RNA oligos were resolved on a 17% polyacrylamide gel containing 7 M Urea in 0.5X TBE and blotted to a positively charged nylon membrane (Hybond-XL, GE LifeSciences) with a Bio-Rad Transblot SD. Membranes were probed with complementary oligonucleotides end-labeled with *γ*^32^P-ATP using OptiKinase (USB Corporation) in Sigma PerfectHyb buffer at 37°C. Final washes of the blots were performed at 37°C with 0.5X SSC, 0.1% SDS. Probe sequences are listed in Additional file 7.

### Sequencing and analysis

sRNAs were purified from total RNA using the PureLink miRNA Isolation kit (Life Technologies) according to the manufacturer’s protocol. Barcoded libraries were then prepared using the SOLiD Total RNA-Seq kit (Life Technologies). Library quality was assessed using a DNA-1000 Agilent chip, quantified via qPCR, pooled and sequenced using the SOLiD-4 chemistry according to the manufacturer’s recommendations. Trimmed sequences corresponding to known miRNA’s and their associated raw counts from 18 sRNA libraries are presented in Additional file 8.

## Abbreviations

CEW, Corn earworm; FAW, Fall armyworm; dsRNA, Double-stranded RNA; GI track, Gastrointestinal track; miRNA, MicroRNAs; NCBI, The National Center for Biotechnology Information; NT, Nucleotide sequence database; siRNA, Small interfering RNA; SRA, The Sequence Read Archive; sRNA, Small RNA; WCR, Western corn rootworm.

## Competing interests

All authors were employed by Monsanto while engaged in the research project described in this publication. The authors declare competing financial interests.

## Authors’ contributions

SI and GH conceived the experiments. YZ did computational analysis. EW did insect feeding experiment and miRNA Northern blotting. SI, GH and YZ wrote the paper. CL and JP edited the manuscript. All authors read and approved the final manuscript.

## Supplementary Material

Additional file 1 **Table S1.**Public sRNA datasets: organism and sample source distribution. Click here for file

Additional file 2 **Table S2.**Public sRNA datasets and presence of plant miRNAs. Click here for file

Additional file 3 **Table S3.**Abundant miRNAs in rice, soy and corn seeds. Click here for file

Additional file 4 **Table S4.**Reads from human datasets map to non-human animal miRNAs. Click here for file

Additional file 5 **Table S5.**Reads from insect libraries map to lettuce-specific sequence. Click here for file

Additional file 6 **Figure S1.**Comparison of plant miRNA abundance between insect and diet source plant tissues sRNA datasets (A, B and C), and between plant miRNAs from all plant libraries and all insect libraries (D) in the run. Click here for file

Additional file 7 Small RNA probes for northern blot analysis. Click here for file

Additional file 8 **Table S6.**sRNA sequences and raw counts from insect feeding experiment parsed against miRBase v17. Click here for file
