# Supplementary material for: Analysis of plant-derived miRNAs in animal small RNA datasets
Source: BMC Genomics. 2012 Aug 8;13:381. doi: 10.1186/1471-2164-13-381 (PMC3462722; doi:10.1186/1471-2164-13-381)
Supplement: Additional file 1 — Table S1.Public sRNA datasets: organism and sample source distribution. [file 1471-2164-13-381-S1.docx]

| **Supplemental table 1.** Public sRNA datasets: organism and sample source distribution | | | |
| --- | --- | --- | --- |
| **Organism** | **Raw reads** | **sRNA datasets** | **organ/culture (number of datasets)** |
| human | 329,566,148 | 14 | cultured cell (8), heart (4), blood (2) |
| monkey | 31,458,389 | 2 | uterus (2) |
| mouse | 344,112,666 | 50 | spleen (14), bone marrow (7), lymph nodes/spleen (5), blood (4), lymph nodes (3), uterus (2), brain (1), cell line (1), embryo stem cell (1), embryonic fibroblasts (1), heart (1), kidney (1), liver (1), lung (1), ovaries (1), pancreas (1), salivary glands (1), skeletal muscle (1), skin (1), testes (1), thymus (1) |
| pig | 67,238,601 | 6 | abdominal fat (2), liver (2), longissimus dorsi muscle (2) |
| chicken | 28,079,770 | 4 | spleen (2), liver (1), blood (1) |
| honeybee | 36,796,459 | 1 | mixed (1) |
| locust | 7,615,181 | 2 | mixed (2) |
| pea aphid | 3,079,932 | 1 | whole insect (1) |
| silkworm | 35,954,658 | 3 | anterior silk gland (1), posterior silk gland (1), whole body (1) |
| **Total** | **883,901,804** | **83** |  |
