# Supplementary material for: Analysis of plant-derived miRNAs in animal small RNA datasets
Source: BMC Genomics. 2012 Aug 8;13:381. doi: 10.1186/1471-2164-13-381 (PMC3462722; doi:10.1186/1471-2164-13-381)
Supplement: Additional file 4 — Table S4.Reads from human datasets map to non-human animal miRNAs. [file 1471-2164-13-381-S4.docx]

Supplemental Table 4. Reads from human datasets map to non-human animal miRNAs

| Sample | Run ID | Source | Raw reads | miRNAs (animal + plant) | Reads map to plant miRNAs | reads map to non-human animal miRNAs* | unique miRNAs | top 3 non-human miRNAs (miRNA ID=number of reads)** |
| --- | --- | --- | --- | --- | --- | --- | --- | --- |
| GSM416753 | SRR029124 | Hela | 4712823 | 942276 |  | 1180 | 87 | dre-miR-27d=251,dre-let-7g=195,xtr-let-7e=92 |
| GSM494809 | SRR039190 | blood | 5401752 | 1175650 | 5342 | 1154 | 115 | dre-miR-107b=252,spu-let-7=121,dre-let-7g=103 |
| GSM494810 | SRR039191 | blood | 3302389 | 2195526 | 4153 | 5839 | 51 | dre-let-7g=4490,dre-miR-107b=383,gga-let-7k=291 |
| GSM755025 | SRR309288 | HeLa | 13823407 | 1610689 | 12 | 2427 | 98 | spu-let-7=417,aga-let-7=326,dre-let-7g=278 |
| GSM755026 | SRR309289 | HeLa | 29875953 | 1725049 | 4 | 2930 | 96 | dre-let-7g=504,spu-let-7=343,pma-let-7c=329 |
| GSM755027 | SRR309290 | HeLa | 23616037 | 1719371 | 5 | 3627 | 102 | spu-let-7=595,dre-let-7g=464,aga-let-7=460 |
| GSM792462 | SRR342894 | heart | 33162427 | 734171 | 1 | 804 | 65 | gga-miR-1b=187,dre-miR-30a=97,dre-miR-148=88 |
| GSM792463 | SRR342895 | heart | 30885544 | 1134583 |  | 788 | 55 | dre-miR-30a=197,gga-miR-1b=102,xtr-miR-24b=58 |
| GSM792464 | SRR342896 | heart | 33375040 | 1491793 | 1 | 1475 | 68 | dre-let-7g=211,pma-let-7d=172,dre-miR-126b=136 |
| GSM792465 | SRR342897 | HeLa | 34060147 | 639017 |  | 457 | 55 | dre-miR-30a=84,gga-miR-1b=50,dre-let-7g=40 |
| GSM792466 | SRR342898 | HeLa | 32298086 | 1475337 |  | 1384 | 77 | dre-miR-30a=120,dre-miR-148=116,gga-miR-1b=111 |
| GSM792467 | SRR342899 | HeLa | 29384411 | 1409757 | 1 | 1000 | 63 | gga-miR-1b=182,dre-miR-30a=148,rno-miR-1=113 |
| GSM792468 | SRR342900 | heart | 28603215 | 1170451 | 3 | 797 | 69 | dre-miR-30a=146,dre-let-7g=62,xtr-miR-27c=53 |
| GSM792469 | SRR342901 | HeLa | 27064917 | 1127925 |  | 790 | 61 | dre-miR-30a=172,gga-miR-1b=101,dre-let-7g=71 |

*reads matching to miRNAs must start from 5' base 1. A Read is counted as a non-human animal miRNA read if its matched non-human animal miRNA is 20 nt or longer, and the read does not have >19 nt long subsequence perfectly matched to the human reference genome.

**dre-let-7g (22nt, from zebrafish) is conserved in Fugu rubripes (fru-let-7g) and Tetraodon nigroviridis (tni-let-7g); gga-miR-1b (21nt, from Gallus gallus) is conserved in Xenopus tropicalis (xtr-miR-1b) and X. laevis (xla-miR-1b); aga-let-7 (21nt, from Anopheles gambiae) is conserved in Aedes aegypti (aae-let-7), Culex quinquefasciatus (cqu-let-7) and Acyrthosiphon pisum (api-let-7); dre-miR-148 (22nt, from zebrafish) is conserved in Fugu rubripes (fru-miR-148) and Tetraodon nigroviridis (tni-miR-148); dre-miR-107b (21 nt, zebrafish), dre-miR-30a (22nt, zebrafish), dre-miR-27d (22nt, zebrafish), spu-let-7 (22nt, Strongylocentrotus purpuratus) and pma-let-7d (22nt, Petromyzon marinus) seem to be species-specific.
