# Supplementary material for: Analysis of plant-derived miRNAs in animal small RNA datasets
Source: BMC Genomics. 2012 Aug 8;13:381. doi: 10.1186/1471-2164-13-381 (PMC3462722; doi:10.1186/1471-2164-13-381)
Supplement: Additional file 5 — Table S5.Reads from insect libraries map to lettuce-specific sequence. [file 1471-2164-13-381-S5.docx]

**Supplemental Table 5.** Reads from insect libraries map to lettuce-specific sequence.

| Sample | Organism | Description | Raw reads | Reads map to lettuce-specific sequence* |
| --- | --- | --- | --- | --- |
| lettuce 1 ~ 10 | Lactuca sativa | summary of 10 lettuce samples | 151,549,912 | 543,228 |
| feeding 1 | WCR | neonate | 16,544,248 | 29 |
| feeding 2 | CEW | neonate | 14,016,629 | 35 |
| feeding 3 | FAW | neonate | 15,429,925 | 24 |
| feeding 4 | CEW | diet corn leaf, carcass, 5^th^ instar, rep1 | 13,469,808 | 11 |
| feeding 5 | CEW | diet corn leaf, carcass, 5^th^ instar, rep2 | 19,188,383 | 39 |
| feeding 6 | CEW | diet corn leaf, carcass, 5^th^ instar, rep3 | 13,020,144 | 14 |
| feeding 7 | FAW | diet corn leaf, carcass, 5^th^ instar, rep1 | 14,496,854 | 18 |
| feeding 8 | FAW | diet corn leaf, carcass, 5^th^ instar, rep2 | 23,688,055 | 64 |
| feeding 9 | FAW | diet corn leaf, carcass, 5^th^ instar, rep3 | 8,346,959 | 12 |
| feeding 10 | FAW | diet soy leaf, carcass, 5^th^ instar, rep1 | 13,583,831 | 38 |
| feeding 11 | FAW | diet soy leaf, carcass, 5^th^ instar, rep2 | 20,007,178 | 92 |
| feeding 12 | FAW | diet soy leaf, carcass, 5^th^ instar, rep3 | 16,090,620 | 16 |
| feeding 13 | WCR | diet corn root, carcass, 3^rd^ instar, rep1 | 23,799,773 | 79 |
| feeding 14 | WCR | diet corn root, carcass, 3^rd^ instar, rep2 | 20,263,174 | 11 |
| feeding 15 | WCR | diet corn root, carcass, 3^rd^ instar, rep3 | 18,023,806 | 37 |
| feeding 16 | CEW | diet soy leaf, carcass, 5^th^ instar, rep1 | 13,292,508 | 37 |
| feeding 17 | CEW | diet soy leaf, carcass, 5^th^ instar, rep2 | 17,506,112 | 42 |
| feeding 18** | CEW | diet soy leaf, carcass, 5^th^ instar, rep3 | 18,634,490 | 0 |

*Run 2 does not have lettuce libraries.

**The library failed in Run 1 and re-sequenced in Run 2. Data shown is from Run 2.
