# Supplementary material for: Analysis of plant-derived miRNAs in animal small RNA datasets
Source: BMC Genomics. 2012 Aug 8;13:381. doi: 10.1186/1471-2164-13-381 (PMC3462722; doi:10.1186/1471-2164-13-381)
Supplement: Additional file 6 — Figure S1.Comparison of plant miRNA abundance between insect and diet source plant tissues sRNA datasets (A, B and C), and between plant miRNAs from all plant libraries and all insect libraries (D) in the run. [file 1471-2164-13-381-S6.docx]

Supplemental Figure 1. Comparison of plant miRNA abundance between insect and diet source plant tissue sRNA datasets (A, B and C), and between plant miRNAs from all plant libraries and all insect libraries (D) in the run.
