# Supplementary material for: Analysis of plant-derived miRNAs in animal small RNA datasets
Source: BMC Genomics. 2012 Aug 8;13:381. doi: 10.1186/1471-2164-13-381 (PMC3462722; doi:10.1186/1471-2164-13-381)
Supplement: Additional file 7 — Small RNA probes for northern blot analysis. [file 1471-2164-13-381-S7.docx]

**Additional file 7.** DNA probes for northern blot analysis

| **miRNA** | **Probe Sequence** |
| --- | --- |
| miR166 | GGGGAATGAAGCCTGGTCCGA |
| miR168 (corn) | GTCCCGATCTGCACCAAGCGA |
| miR168 (soy) | TTCCCGACCTGCACCAAGCGA |
| miR-279 (CEW) | TGGATGAGTGTGGATCTAGTCA |
| miR-307 (FAW) | TCGCTCACTCAAGGAGGTTGTGA |
| miR-8-5p (WCR) | TCTAATGCTGCCCGGTAAGATG |
